# Genistein Up-Regulates Tumor Suppressor MicroRNA-574-3p in Prostate Cancer

**DOI:** 10.1371/journal.pone.0058929

**Published:** 2013-03-12

**Authors:** Takeshi Chiyomaru, Soichiro Yamamura, Shinichiro Fukuhara, Hideo Hidaka, Shahana Majid, Sharanjot Saini, Sumit Arora, Guoren Deng, Varahram Shahryari, Inik Chang, Yuichiro Tanaka, Z. Laura Tabatabai, Hideki Enokida, Naohiko Seki, Masayuki Nakagawa, Rajvir Dahiya

**Affiliations:** 1 Department of Urology, San Francisco Veterans Affairs Medical Center and University of California San Francisco, San Francisco, California, United States of America; 2 Department of Urology, Graduate School of Medical and Dental Sciences, Kagoshima University, Kagoshima, Japan; 3 Department of Pathology, San Francisco Veterans Affairs Medical Center and University of California San Francisco, San Francisco, California, United States of America; 4 Department of Functional Genomics, Graduate School of Medicine, Chiba University, Chiba, Japan; University of Toronto, Canada

## Abstract

Genistein has been shown to inhibit cancers both in vitro and in vivo, by altering the expression of several microRNAs (miRNAs). In this study, we focused on tumor suppressor miRNAs regulated by genistein and investigated their function in prostate cancer (PCa) and target pathways. Using miRNA microarray analysis and real-time RT-PCR we observed that miR-574-3p was significantly up-regulated in PCa cells treated with genistein compared with vehicle control. The expression of miR-574-3p was significantly lower in PCa cell lines and clinical PCa tissues compared with normal prostate cells (RWPE-1) and adjacent normal tissues. Low expression level of miR-574-3p was correlated with advanced tumor stage and higher Gleason score in PCa specimens. Re-expression of miR-574-3p in PCa cells significantly inhibited cell proliferation, migration and invasion in vitro and in vivo. miR-574-3p restoration induced apoptosis through reducing Bcl-xL and activating caspase-9 and caspase-3. Using GeneCodis software analysis, several pathways affected by miR-574-3p were identified, such as ‘Pathways in cancer’, ‘Jak-STAT signaling pathway’, and ‘Wnt signaling pathway’. Luciferase reporter assays demonstrated that miR-574-3p directly binds to the 3′ UTR of several target genes (such as RAC1, EGFR and EP300) that are components of ‘Pathways in cancer’. Quantitative real-time PCR and Western analysis showed that the mRNA and protein expression levels of the three target genes in PCa cells were markedly down-regulated with miR-574-3p. Loss-of-function studies demonstrated that the three target genes significantly affect cell proliferation, migration and invasion in PCa cell lines. Our results show that genistein up-regulates tumor suppressor miR-574-3p expression targeting several cell signaling pathways. These findings enhance understanding of how genistein regulates with miRNA in PCa.

## Introduction

The most commonly diagnosed type of cancer among men in 2012 is prostate cancer (PCa) that is expected to account for 29% (241,740) of all new cancer cases. PCa ranks second to lung cancer in cancer-related deaths and is expected to account for 9% (28,170) of all male cancer deaths in 2012 [1]. Metastatic PCa is not curable and continues to be the major cause of cancer deaths [2]. Palliation can be achieved by hormone deprivation therapy however after an excellent initial response, in approximately 2 to 3 years most of these PCas will relapse to the castration resistant form of the disease [3] with death usually occurring within several years [4]. There are no successful treatments for androgen-independent PCa. A better understanding of biological mechanisms of androgen-independent PCa may lead to novel approaches to treat unresponsive PCa more successfully.

Development of chemotherapeutic agents with low patient toxicity is currently being investigated by many scientists. Many of these agents are derived from natural plant products. Genistein is a phytoestrogenic isoflavonoid that has pleiotropic biological effects in a wide variety of cancers without any visible toxicity to normal cells [5]. Genistein is a protein tyrosine kinase inhibitor and affects cell proliferation, apoptosis, tumor angiogenesis, metastasis and attenuates multidrug resistance involving key components of signal transduction pathways [6], [7], [8].

microRNAs (miRNAs) are small non-coding RNAs (21–23 nucleotides) that mainly bind imperfectly to the 3′ untranslated region (UTR) of target mRNAs and negatively regulate gene expression post-transcriptionally by translational repression and degradation of target mRNA [9], [10]. Since the identification of miRNAs in 1993 over 1500 human miRNAs have been registered in the miRBase database (http://microrna.sanger.ac.uk/). Bioinformatics indicate that more than 60% of protein-coding genes may be targeted by miRNAs [11]. miRNAs play an important part in many biological processes, such as development, differentiation, proliferation, apoptosis, angiogenesis and metabolism. In addition, they are key regulators in many diseases including cancer [12] and miRNAs may function as oncogenes or tumor suppressor genes [13], [14]. The effects of genistein on the regulation of several miRNAs have been reported [15], [16], [17]. Our laboratory has shown that genistein inhibits cancer cell growth targeting oncogenic miRNAs such as miR-21, miR-151, miR-221 and miR-222. In this study, we focused on tumor suppressor miR-574-3p that is regulated by genistein and investigated its function in PCa and target pathways.

## Results

### Genistein Treatment Increases miR-574-3p Expression which is Down-regulated in PCa

**Figure 1:**
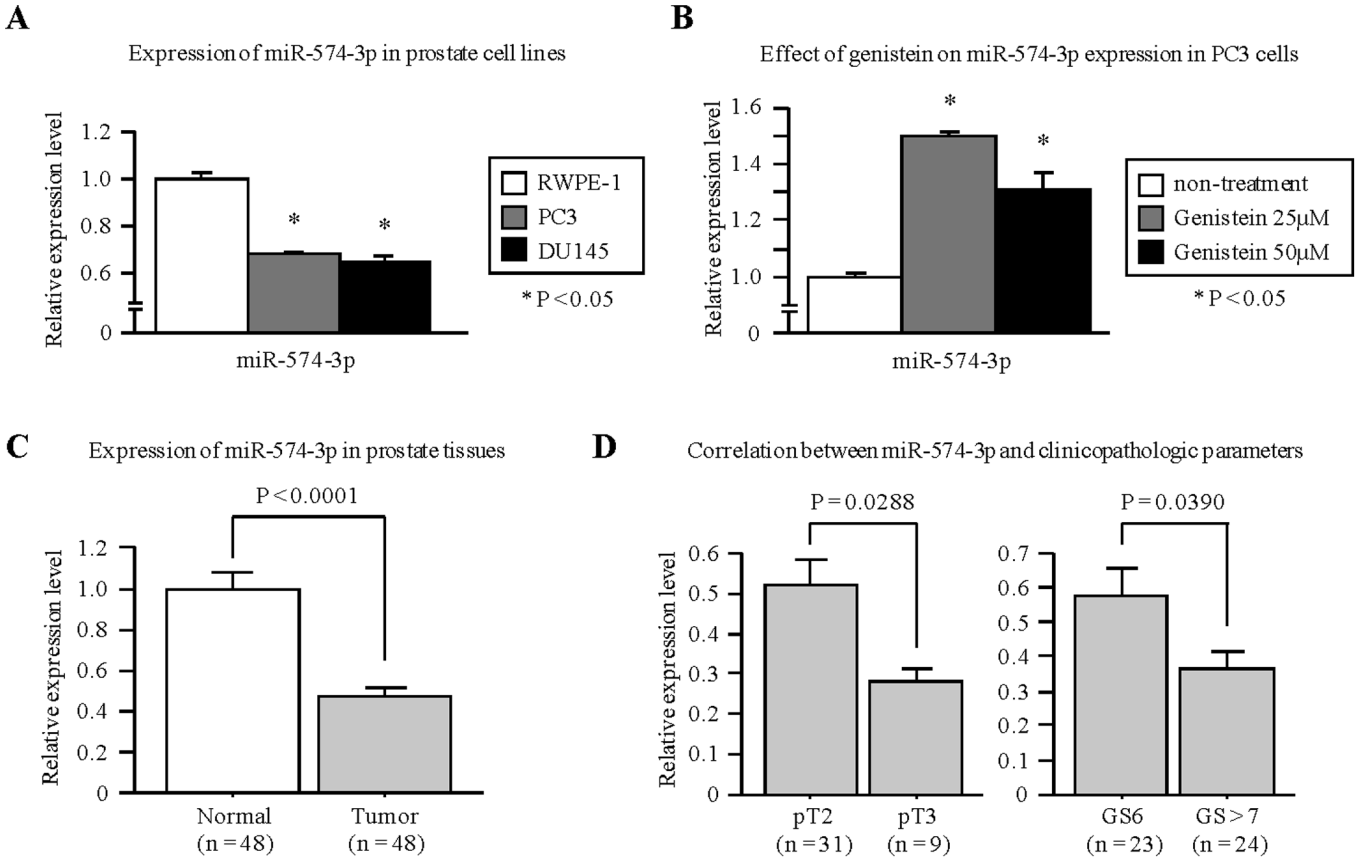
Effect of genistein treatment and expression of miR-574-3p in PCa cells and specimens. (A) Expression of miR-574-3p in PCa cell lines (DU145 and PC3) and normal prostate epithelial cells (RWPE-1). Real-time PCR showed that the expression levels of miR-574-3p were down-regulated in PCa cell lines (DU145 and PC3). miR-574-3p expression was normalized to RNU48. Data are presented as mean ± SE. *, P<0.05. (B) Expression levels of miR-574-3p after treatment with genistein (25 µM and 50 µM). miR-574-3p expression increased by 30–50% in genistein treated cells compared with controls. *, P<0.05. (C) miR-574-3p expression in clinical samples (Adjacent normal tissue, n = 48; PCa, n = 48). miR-574-3p expression was determined by real-time PCR and normalized to RNU48. P<0.0001. (D) Real-time PCR showing correlation of clinicopathological characteristics with miR-574-3p expression.

To determine relative expression levels of miR-574-3p in prostate cells, we performed quantitative real-time PCR using PC3 and DU145 cell lines and compared them with normal prostate epithelial cells (RWPE-1). We observed that miR-574-3p expression was significantly down-regulated in PCa cell lines compared to RWPE-1 cells (PC3 0.68-fold, DU145 0.65-fold) (Fig. 1A).

To identify miRNAs regulated by genistein, we conducted a miRNA microarray using PC3 cells after genistein treatment (Table 1). The expression of 33 miRNAs was significantly up-regulated using two concentrations of genistein (25 µM and 50 µM) with miR-574-3p being the most affected. Previous miRNA expression studies from our lab also showed that miR-574-3p was significantly down-regulated in PCa samples compared with non-cancerous prostate tissues [18]. To confirm expression of miR-574-3p, we performed TaqMan quantitative real-time PCR analysis and observed that miR-574-3p expression was significantly up-regulated with genistein treatment (1.31 to 1.50-fold) (Fig. 1B).

**Table 1:** Effect of genistein treatment for microRNA profiles in prostate cancer cells (PC3).

	Normalized Intensity			Ratio			P value	
	non-treat	25 µM	50 µM	25 µM/non	50 µM/non	Average	25 µM	50 µM
hsa-miR-574-3p	291.88	825.70	794.68	2.83	2.72	2.78	0.0000629	0.0000743
hsa-miR-29a	1839.50	3294.02	5835.99	1.79	3.17	2.38	0.0008651	0.0000297
hsa-miR-29b	1197.37	1878.11	4315.52	1.57	3.6	2.38	0.0031300	0.0000131
hsa-miR-1234	253.86	805.18	439.51	3.17	1.73	2.34	0.0000002	0.0000181
hsa-miR-4700-3p	190.06	474.90	346.86	2.5	1.83	2.14	0.0000049	0.0000504
hsa-miR-4732-3p	162.91	387.93	307.66	2.38	1.89	2.12	0.0001546	0.0001308
hsa-miR-5096	166.98	493.47	250.64	2.96	1.5	2.11	0.0000680	0.0090343
hsa-miR-1972	149.33	258.95	378.93	1.73	2.54	2.1	0.0000051	0.0000303
hsa-miR-3663-3p	162.91	343.96	323.10	2.11	1.98	2.05	0.0000210	0.0001064
hsa-miR-3194-5p	166.98	341.03	329.04	2.04	1.97	2.01	0.0000023	0.0001121
hsa-miR-4436b-5p	362.47	941.99	538.10	2.6	1.48	1.96	0.0000694	0.0012833
hsa-miR-3646	156.12	294.13	310.03	1.88	1.99	1.93	0.0000777	0.0000856
hsa-miR-3613-3p	153.40	364.48	239.95	2.38	1.56	1.93	0.0000190	0.0012331
hsa-miR-1203	115.39	220.84	217.38	1.91	1.88	1.9	0.0000648	0.0006706
hsa-miR-3679-3p	108.60	201.30	192.43	1.85	1.77	1.81	0.0004517	0.0021585
hsa-miR-1281	859.34	1674.86	1443.26	1.95	1.68	1.81	0.0000875	0.0010415
hsa-miR-4640-3p	153.40	297.06	244.70	1.94	1.6	1.76	0.0000244	0.0004460
hsa-miR-2467-3p	203.63	330.28	369.43	1.62	1.81	1.72	0.0006034	0.0000831
hsa-miR-484	224.00	428.00	320.72	1.91	1.43	1.65	0.0000459	0.0007682
hsa-miR-466	308.17	545.26	472.77	1.77	1.53	1.65	0.0002347	0.0016229
hsa-miR-29c	366.54	521.81	686.59	1.42	1.87	1.63	0.0007586	0.0001604
hsa-miR-1909*	107.25	170.03	174.62	1.59	1.63	1.61	0.0015811	0.0012480
hsa-miR-4440	177.84	259.93	313.60	1.46	1.76	1.61	0.0003777	0.0004528
hsa-miR-4252	131.68	188.59	226.88	1.43	1.72	1.57	0.0021412	0.0004876
hsa-miR-4312	207.71	322.46	327.85	1.55	1.58	1.57	0.0006465	0.0017571
hsa-miR-3189-5p	244.36	352.76	411.00	1.44	1.68	1.56	0.0000139	0.0000392
hsa-miR-4720-5p	122.18	193.48	186.50	1.58	1.53	1.55	0.0005705	0.0002532
hsa-miR-1226	154.76	241.36	237.57	1.56	1.54	1.55	0.0011385	0.0046299
hsa-miR-3619-3p	173.77	273.61	258.95	1.57	1.49	1.53	0.0001530	0.0014201
hsa-miR-4274	153.40	222.79	243.51	1.45	1.59	1.52	0.0028863	0.0030237
hsa-miR-760	271.51	421.16	396.75	1.55	1.46	1.51	0.0005929	0.0003805
hsa-miR-214	135.76	209.11	192.43	1.54	1.42	1.48	0.0042072	0.0048627
hsa-miR-3935	139.83	199.34	212.63	1.43	1.52	1.47	0.0000086	0.0050444

### miR-574-3p is Significantly Down-regulated in PCa Tissue Specimens

We evaluated expression levels of miR-574-3p in human PCa tissues (n = 48) and adjacent non-cancerous tissues (n = 48). The expression level of miR-574-3p was significantly lower in PCa compared with normal tissues (P<0.0001; Fig. 1C). To determine if the levels of miR-574-3p in tumor tissues correlates with clinic-pathological factors, we analyzed miR-574-3p expression levels in human tumor samples. Clinical demographics of the study cohort are summarized in Table 2. Correlation of 574-3p expression with clinicopathological variables such as pathological stage (pT) and Gleason score is shown in Fig. 1D. These results reveal that cases with low miR-574-3p expression increase from low grade, low pathological stage to high grade and high pathological stage. These results suggest that miR-574-3p is significantly down-regulated in PCa and may be a putative tumor suppressor in PCa.

**Table 2:** Prostate cancer patient information.

Characteristics			(%)
Age (years)			
Median (range)	62 (47–81)		
PSA (ng/ml)			
Median (range)	7.0 (0.2–90)		
Total number	48		(100.0)
Gleason Score			
GS 6	23		(47.9)
GS 7	15		(31.3)
GS 8	6		(12.5)
GS 9	3		(6.2)
unknown	1		(2.1)
Pathological tumor stage			
pT2a	8		(16.7)
pT2b	10		(20.8)
pT2c	13		(27.1)
pT3a	8		(16.7)
pT3b	1		(2.1)
unknown	8		(16.7)

Abbreviations: PSA = prostate-specific antigen; GS = Gleason Score.

### Effect of miR-574-3p Over-expression on Cell Proliferation, Migration, and Invasion in PCa Cell Lines in vitro and in vivo

**Figure 2:**
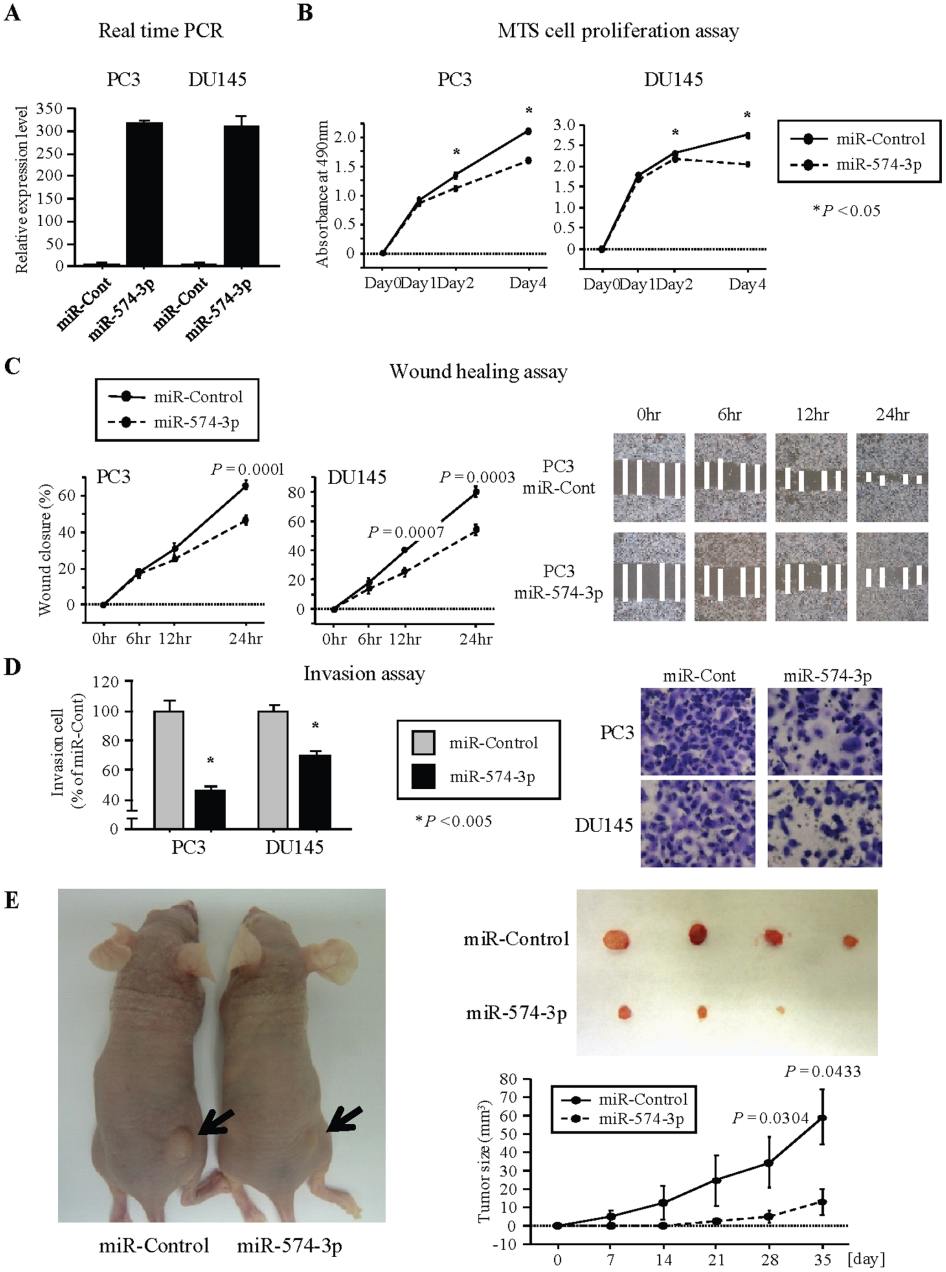
Effect of miR-574-3p overexpression on PCa cell proliferation, migration and invasion in vitro and in vivo. (A) miR-574-3p expression levels in PCa cell lines (PC3 and DU145) were determined by real-time PCR at 72 hours after transfection of Pre-miR miRNA precursor. miR-574-3p expression was normalized to RNU48. Data are presented as the mean ± SE. (B) Overexpression of miR-574-3p significantly inhibits cell viability. Cell viability was analyzed by the MTS cell proliferation assay 1, 2 and 4 days after transient transfection. *, P<0.05. (C) Over-expression of miR-574-3p significantly inhibits cell migration. After transfection (48 hours), a wound was formed by scraping and measured after 6, 12 and 24 hours. Representative images of wound healing assay are shown at 200× magnification. **, P<0.0001. *, P<0.005. (D) Over-expression of miR-574-3p significantly decreased cell invasion. Representative images of invasion assay are shown at 200× magnification. *, P<0.005. (E) Representative images of tumors in nude mice 5 weeks after subcutaneous injection of transfected miR-574-3p DU145 cell lines or control cell lines and time course of tumor growth.

To examine the functional roles of miR-574-3p, we performed gain-of-function studies using a pre-miR-574-3p miRNA precursor transfected into PC3 and DU145 cells. The expression of miR-574-3p was markedly up-regulated in Pre-miR miRNA precursor transfectants (Fig. 2A; PC3 316.8-fold, DU145 307.5-fold). Cell proliferation assay (MTS) and wound healing assay showed significant inhibition in miR-574-3p transfectants in both the PC3 and DU145 cells compared to the control transfectants (Fig. 2B and 2C). Invasion assay (Matrigel) also showed that the number of invading cells was significantly decreased in miR-574-3p transfectants compared with their counterparts (Fig. 2D). To confirm the effect of miR-574-3p on tumorigenicity in vivo, miR-574-3p and miR-control-transfected DU145 cells were subcutaneously injected into nude mice. We observed that miR-574-3p over-expression inhibited DU145 cell tumor formation in vivo (Fig. 2E). These results suggest that miR-574-3p plays an important role in tumor cell progression.

### miR-574-3p Influences Cellular Apoptosis in PCa Cells

**Figure 3:**
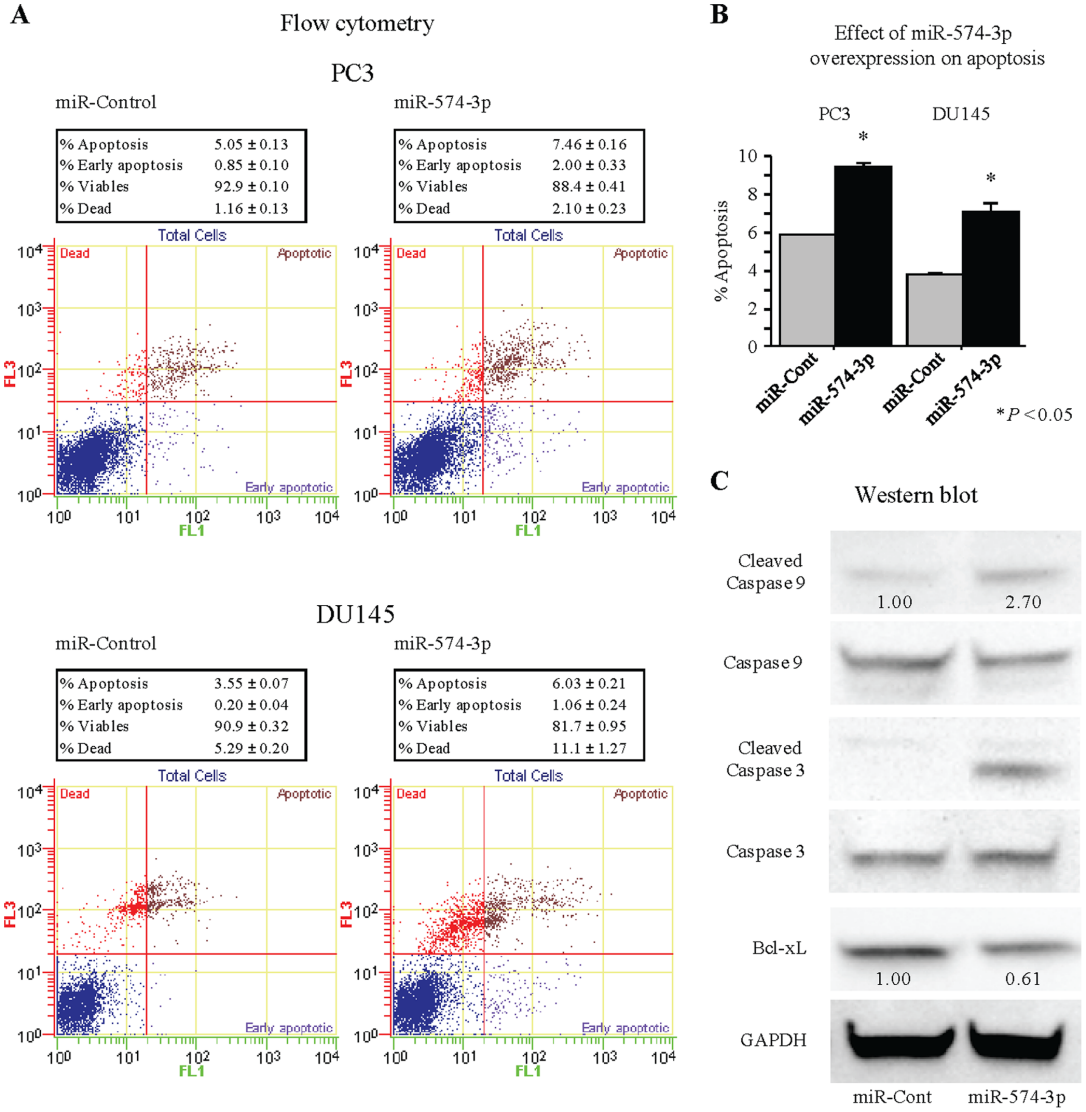
Effect of miR-574-3p overexpression on apoptosis. (A) Apoptosis assay using flow cytometry. Representative quadrant figures of miR-control and miR-574-3p transfectants in PC3 (upper) and DU145 (lower) cells. (B) The bar chart indicates the ratio of apoptotic cell fractions (early apoptotic plus apoptotic cells) in miR-574-3p transfectants compared with controls. Data for the apoptotic cell fractions are expressed as the relative value for the average expression of the miR-control transfectant. *, P<0.05. (C) Immunoblots analysis for apoptotic markers in miR-control and miR-574-3p transfected DU145 cells. GAPDH was used as a loading control.

Since miR-574-3p restoration significantly inhibited cell proliferation in PCa cell lines, we hypothesized that its restoration may induce apoptosis. Fig. 3A and 3B showed that the apoptotic and early apoptotic fractions (upper right and lower right in the quadrant images, respectively) were greater in miR-574-3p transfectants compared to control. This points to a pro-apoptotic role for miR-574-3p and suggests that it affects apoptotic pathways and regulates tumorigenicity. Therefore we examined the expression of various apoptotic proteins by Western blot analysis and found that miR-574-3p re-expression causes Bcl-xL down-regulation (Fig. 3C). Also, cleaved caspases-9 and -3 were up-regulated (Fig. 3C), further supporting the fact that miR-574-3p is a pro-apoptotic miRNA.

### Search for miR-574-3p Target Genes by in silico Analysis

To search for putative target genes of miR-574-3p, we used the TargetScan database. This program showed that miR-574-3p has 437 predicted target genes. We used in silico analysis to identify the biological processes or pathways potentially regulated by miR-574-3p. The candidate target genes were assigned to pathways using GeneCodis software analysis (http://genecodis.cnb.csic.es) [19], [20], [21], and statistically enriched pathways were identified such as ‘Pathways in cancer’, ‘Jak-STAT signaling pathway’, and ‘Wnt signaling pathway’ (P<0.05, Table 3). We then performed gene expression analyses for all candidate target genes involved in each of the 9 pathways using microarray expression data, which were approved by the Gene Expression Omnibus (GEO) and focused on the ‘Pathways in cancer’. From this analysis, several crucial gene targets were identified, including tropomyosin 3 (TPM3), wingless-type MMTV integration site family, member 5A (WNT5A), ras-related C3 botulinum toxin substrate 1 (rho family, small GTP binding protein Rac1) (RAC1), epidermal growth factor receptor (EGFR), retinoid X receptor, alpha (RXRA), v-akt murine thymoma viral oncogene homolog 2 (AKT2), and E1A binding protein p300 (EP300).

**Table 3:** Pathways regulated by the putative target genes of miR-574-3p.

Number of Genes	Pathway ID	KEGG Pathway	p-value	Gene Symbol
13	(KEGG) 05200	Pathways in cancer	0.0122	IL6,COL4A4,KITLG,CUL2,WNT7B,
				BIRC5,TPM3,EP300,EGFR,WNT5A,
				RAC1,RXRA,AKT2
10	(KEGG) 04020	Calcium signaling pathway	0.0112	CAMK2B,ATP2A2,CAMK2A,HTR7,
				PTGER3,GNAQ,SLC25A4,EGFR,
				PTAFR,ATP2B3
10	(KEGG) 04060	Cytokine-cytokine receptor interaction	0.0307	IL6,KITLG,IL28RA,BMPR2,EGFR,
				LEP,OSMR,ACVR2B,ACVR1B,IL2RB
9	(KEGG) 05016	Huntington's disease	0.0171	NDUFA4L2,CLTC,POLR2L,POLR2E,
				CREB5,EP300,GNAQ,SLC25A4,HIP1
8	(KEGG) 04630	Jak-STAT signaling pathway	0.0174	IL6,IL28RA,SOCS4,EP300,LEP,
				OSMR,IL2RB,AKT2
8	(KEGG) 04310	Wnt signaling pathway	0.0175	PRICKLE2,CAMK2B,WNT7B,
				CAMK2A,EP300,WNT5A,RAC1,NKD1
7	(KEGG) 04916	Melanogenesis	0.0172	CAMK2B,KITLG,WNT7B,CAMK2A,
				EP300,GNAQ,WNT5A
7	(KEGG) 04360	Axon guidance	0.0245	EPHA8,DPYSL2,ABLIM2,PLXNA2,
				RAC1,RGS3,EPHB3
5	(KEGG) 04664	Fc epsilon RI signaling pathway	0.0456	MAPK11,SYK,RAC1,LAT,AKT2

### Luciferase Reporter Assays Using Vectors Containing 3′UTR Binding Sites of Putative Target Genes

**Figure 4:**
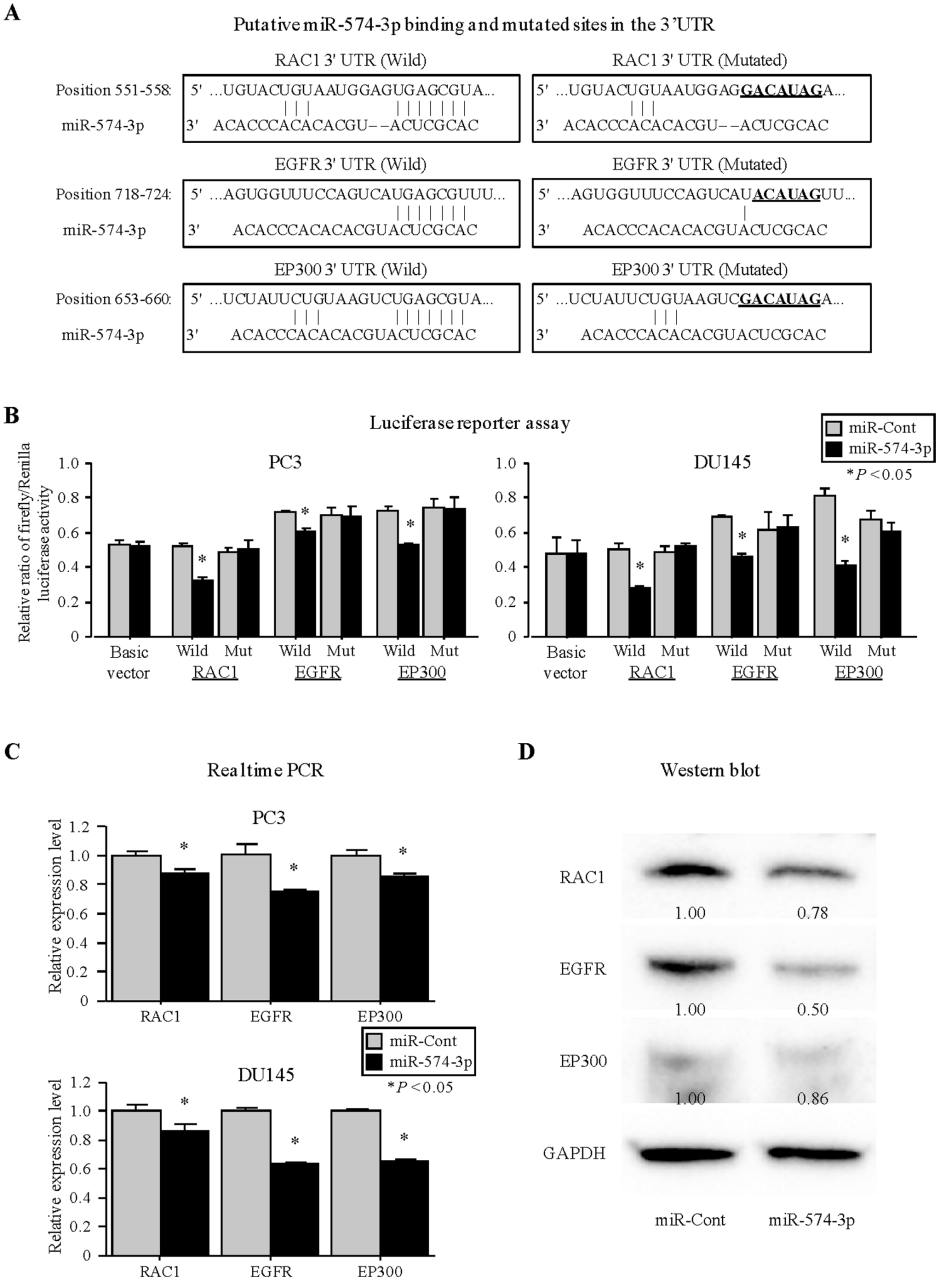
miR-574-3p targets RAC1, EGFR, EP300. (A) Putative miR-574-3p binding and mutated sites in the 3′UTR of target genes. (B) Luciferase reporter assays using vectors encoding putative 3′UTR binding sites. PC3 and DU145 cells were transiently transfected with Pre-miR miRNA precursor or negative control, followed by transient transfection with basic vector or wild-type 3′UTR reporter plasmids or mutated 3′UTR plasmids for 24 hours. 3′UTR reporter activity was measured by luciferase assay and normalized to the activity of Renilla luciferase. Data are presented as the mean ± SE. *, P<0.05. (C). The mRNA levels of the three target genes of miR-574-3p were determined by quantitative real-time PCR analyses after transfection with miR-574-3p mimics and negative control in PCa cell lines (PC3 and DU145). *, P<0.05. (D) Immunoblot analysis for target genes in miR-control and miR-574-3p transfected PC3 cells. GAPDH was used as a loading control.

To confirm the binding of miR-574-3p to the 3′ UTR of these 7 target genes, we performed luciferase reporter assays. Each target has one predicted binding site for miR-574-3p (Fig. 4A). We cloned the putative miR-574-3p 3′UTRs target into a luciferase reporter assay vector. Luciferase reporter assays demonstrated that miR-574-3p decreased the relative luciferase activities of RAC1, EGFR and EP300 (Fig. 4B) except for other four genes. Mutation of the putative miR-574-3p binding sites in these 3′UTRs decreased the response to miR-574-3p indicating that miR-574-3p binds directly to the 3′UTRs of RAC1, EGFR and EP300.

### Regulation of Target Gene Expression in PCa Cell Lines by miR-574-3p

Quantitative real-time PCR analysis showed that the mRNA expression levels of the three target genes in PC3 and DU145 were repressed in the miR-574-3p transfectants compared with the controls (Fig. 4C). The protein expression levels of the three target genes were also decreased in the miR-574-3p transfectants compared with the controls (Fig. 4D).

### Effect of Target Gene siRNA Knockdown on Cell Proliferation, Migration, and Invasion Activity in PCa Cell Lines

**Figure 5:**
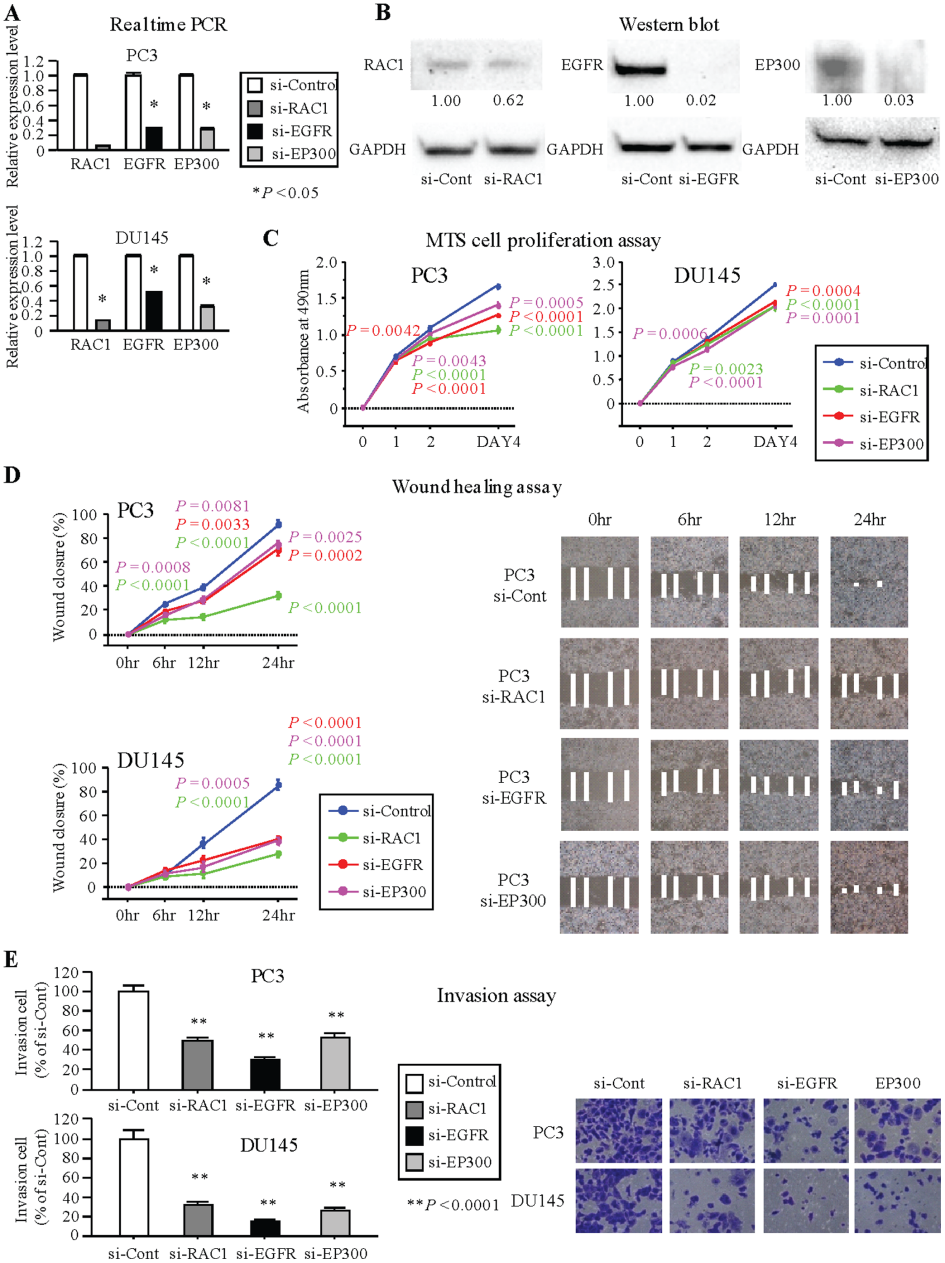
siRNA knockdown of miR-574-3p target genes and effect on PCa cell viability. (A) Target gene expression levels in PCa cell lines (PC3 and DU145) were determined by real-time PCR at 72 hours after transfection of siRNA. Target gene expression was normalized to GAPDH. Data are presented as the mean ± SE. *, P<0.05. (B) Target gene expression in PC3 cell lines were determined by immunoblot analysis at 72 hours after transfection of siRNA. GAPDH was used as a loading control. (C) Knockdown of RAC1, EGFR and EP300 significantly inhibits cell viability. Cell viability was analyzed by the MTS cell proliferation assay 1, 2 and 4 days after transient transfection. (D) Knockdown of RAC1, EGFR and EP300 significantly inhibits cell migration. After transfection (48 hours), a wound was formed by scraping and measured after 6, 12 and 24 hours. Representative images of wound healing assay are shown at 200× magnification. (E) Knockdown of RAC1, EGFR and EP300 significantly decreased cell invasion. Representative images of invasion assay are shown at 200× magnification. **, P<0.0001.

To examine the functional role of the target genes, we performed loss-of-function studies using si-RNA knockdown with PC3 and DU145 cells. The mRNA and protein expression of RAC1, EGFR and EP300 were markedly repressed in si-RNA transfectants compared to controls (Fig. 5A and 5B). Cell proliferation assay (MTS) and wound healing assay showed significant inhibition in si-RAC1, si-EGFR and si-EP300 transfectants in both PC3 and DU145 cells compared to the control transfectants (Fig. 5C and 5D). Invasion assay (Matrigel) also showed that the number of invading cells was significantly decreased in si-RAC1, si-EGFR and si-EP300 transfectants compared with their control counterparts (Fig. 5E).

## Discussion

Many studies have shown that genistein regulates cancer cell proliferation, invasion, angiogenesis and metastasis by targeting several genes and signaling pathways [6], [7], [22]. For example genistein inhibited cell invasion reducing expression of MMP2 and MMP9 in human prostate epithelial and metastatic cells where tumor progression is positively correlated with the expression of MMP [23], [24], [25]. We have previously shown that genistein down-regulated the expression of MCM2 by increasing miR-1296 expression in PCa cells [26]. We have also reported that genistein down-regulated miR-221/222 expression in PCa cells resulting in increased expression of tumor suppressor gene ARHI which is target of miR-221/222 [16]. Genistein has also been reported to inhibit PCa angiogenesis by suppression of VEGF-mediated autocrine and paracrine signaling pathways between tumor cells and vascular endothelial cells [27]. The EP300 gene has been identified as a co-activator of HIF1A and plays a role in stimulation of hypoxia-induced genes such as VEGF [28]. RAC1 is also an important regulator of VEGF-mediated angiogenesis [29] and miR-151 has an oncogenic function regulating activation of RAC1 by targeting ARHGDIA [30]. We also recently demonstrated that genistein inhibited PCa cell migration and up-regulated several tumor suppressor genes including ARHGDIA targeted by miR-151. In this study, we found that genistein up-regulated miR-574-3p expression in PCa cells. In silico analysis and luciferase reporter assays demonstrated that RAC1 and EP300 were putative target genes for miR-574-3p. Quantitative real-time PCR and Western analysis showed that mRNA and protein expression levels of RAC1 and EP300 in PCa cells were markedly down-regulated by miR-574-3p. Therefore genistein may down-regulate RAC1 and EP300 by up-regulating miR-574-3p.

Over-expression of Notch1 leads to induction of the EMT phenotype and increased expression of miR-21 [31]. Genistein has been shown to inactivate Notch and hedgehog signaling [31], [32] and we previously reported that genistein inhibited tumor cell growth by reducing miR-21 expression in renal cell carcinoma [15]. Thus genistein has a tumor suppressor function, regulating ‘Notch signaling’ by down-regulation of miR-21. Genistein can also reduce cell proliferation by regulating ‘Wnt signaling’ [33], [34], [35] through miR-574-3p in cancer. In this study EGFR, a putative target gene for miR-574-3p, was up-regulated in PCa and increased in advanced cancer [36], [37]. EGFR expression was correlated with a high Gleason score, disease relapse and hormone-refractory status [37], [38]. Researchers have reported that EGFR is regulated by several miRNAs such as miR-7, miR-128b, miR-133, miR-145, miR146a, miR-146b-5p, miR-331-3p, miR-542-5p [39]–[47]. Genistein up-regulated miR-146a expression in pancreatic cancer cells and functions as a tumor suppressor in castration-resistant PCa [44], [45]. Genistein might down-regulated EGFR levels by up-regulating miR-574-3p.

Genistein induces apoptosis by regulating intrinsic and extrinsic signaling pathways. Pro- and anti-apoptotic Bcl-2 family proteins play a crucial role in regulating the mitochondrial apoptotic pathway [48] and down-regulation of Bcl-xL by genistein induces apoptosis in PCa cells [49]. In this pathway activated caspase-9 accelerates executioner caspase activation, including caspase-3, and successively cleaves signaling molecules and cellular proteins [49], [50]. In our study, miR-574-3p induced apoptosis and regulated expression of Bcl-xL, caspase-9 and caspase-3. Therefore this study shows that genistein induced apoptosis in PCa cells occurs by increased miR-574-3p expression.

In this study, we focused on miR-574-3p that was up-regulated by genistein and was a significantly down-regulated miRNA specific to PCa in the miRNA profile [18]. Our previous studies indicated that miR-574-3p might be a tumor suppressor miRNA in PCa and bladder cancer [18], [51], [52]. miR-574-3p is located on chromosome 4p14, a frequently deleted chromosomal region in PCa and bladder cancer cell lines [51], [53]. Su et al reported that the expression of miR-574-3p was reduced in gastric cancer and cell proliferation, migration and invasion were significantly inhibited in miR-574-3p-transfected gastric cancer cells [54]. They found the CUL2 gene to be a target of miR-574-3p using by computational prediction and experimental validation. Our previous study demonstrated that miR-574-3p has tumor suppressor function and that the oncogenic MESDC1 gene is targeted by miR-574-3p in bladder cancer [52]. In this study, we have demonstrated that miR-574-3p is down-regulated in clinical PCa samples and androgen-independent PCa cell lines (PC3 and DU145). Down-regulation of miR-574-3p expression in tumors is related to high tumor stage and Gleason score indicating that miR-574-3p may be used as a biomarker for tumor progression in PCa.

In conclusion, our results show that genistein up-regulates miR-574-3p expression which targets several cell signaling pathways. These findings enhance our understanding of how genistein regulates miRNA expression in PCa.

## Materials and Methods

### Clinical Prostate Specimens

All tissue slides were reviewed by a board certified pathologist for the identification of PCa foci as well as adjacent normal glandular epithelium. All cancer patients had elevated levels of prostate specific antigen (PSA) and had undergone radical prostatectomy from 1998 to 2004. The patient’ demographics are shown in Table 2. Written informed consent was obtained from all patients and the study was approved by the UCSF Committee on Human Research (Approval number: H9058-35751-01).

### Cell Culture

Human PCa cell lines, PC3 and DU145 and a non-malignant epithelial prostate cell line, RWPE-1, were purchased from The American Type Culture Collection (Manassas, VA, USA). PCa cell lines were cultured in RPMI 1640 medium supplemented with 10% fetal bovine serum (FBS) in a humidified atmosphere of 5% CO2 and 95% air at 37°C. RWPE-1 cells were cultured in keratinocyte growth medium supplemented with 5 ng/mL human recombinant epidermal growth factor and 0.05 mg/mL bovine pituitary extract (Invitrogen, Carlsbad, CA, USA). Subconfluent cells (60%–70% confluent) were treated with genistein (25 µmol/L and 50 µmol/L; Sigma, St Louis, MO, USA) dissolved in dimethylsulfoxide and cells treated with vehicle (dimethylsulfoxide) served as control. Media and genistein were changed every day and cells were grown for 4 days.

### RNA Extraction

RNA was extracted from FFPE human samples using a miRNeasy formalin-fixed paraffin-embedded kit (Qiagen, Valencia, CA, USA) after microdissection. To digest DNA, the Qiagen RNase-Free DNase kit was used. Total RNA was also extracted from PCa cell lines and a non-malignant epithelial prostate cell line using a miRNeasy mini kit (Qiagen) according to the manufacturer’s instructions.

### microRNA Microarray

For miRNA microarray, total RNA was extracted from PC3 cells treated with genistein using a miRNeasy Mini Kit. The miRNA microarray analysis was carried out and analyzed by a commercial company (Phalanx Biotech, Belmont, CA, USA) using the human v3 miRNA OneArray platform that is designed to contain 100% of miRBase Sequence Database Release 17.0.

### Quantitative Real-time PCR

Extracted total RNA was reverse transcribed into single-stranded cDNA using an iScript cDNA Synthesis Kit (Bio-Rad, Hercules, CA, USA) and a TaqMan MicroRNA Reverse Transcription Kit (Applied Biosystems, Foster City, CA, USA). Quantitative real-time PCR analysis was performed with an Applied Biosystems Prism7500 Fast Sequence Detection System using TaqMan universal PCR master mix according to the manufacturer’s protocol (Applied Biosystems). Levels of RNA expression were determined using the 7500 Fast System SDS software version 1.3.1 (Applied Biosystems). PCR parameters for cycling were as follows: 95°C for 20 seconds, 40 cycles of PCR at 95°C for 3 seconds, and 60°C for 30 seconds. All reactions were done in a 10-µL reaction volume in triplicate. The data were analyzed with the delta-delta Ct method to calculate the fold-change. TaqMan probes and primers for RAC1 (assay ID: Hs01902432_s1), EGFR (assay ID: Hs01076078_m1), EP300 (assay ID: Hs00914223_m1), GAPDH (assay ID: Hs02758991_g1), miR-574-3p (assay ID: 002349), RNU48 (Assay ID: 001006) were obtained from Applied Biosystems. GAPDH and RNU48 were used as internal controls.

### Western Analysis

At 72 hours after transfection, cells were lysed with RIPA buffer (Pierce, Brebieres, France) containing protease inhibitors (Sigma). Protein quantification was done using a BCA protein assay kit (Pierce). Protein lysate (30 µg) was separated on 4% to 20% SDS polyacrylamide gels and transferred to a PVDF membrane. Antibodies to EP300 and Bcl-xL were purchased from Invitrogen and Santa Cruz Biotechnology (Santa Cruz, CA, USA). Antibodies against RAC1, EGFR and GAPDH were purchased from GeneTex (Irvine, CA, USA) respectively. Antibodies to cleaved caspase-3, -9 and caspase-3, -9 were purchased from Cell Signaling Technology (Danvers, MA, USA). After incubation with primary antibody the membrane was washed and then incubated with secondary antibodies conjugated to horseradish peroxidase (Cell Signaling Technology, Danvers, MA, USA). Specific complexes were visualized with an echochemiluminescence (ECL) detection system (GE Healthcare, Little Chalfont, UK). The membrane was stripped using ReBlot Plus Strong Antibody Stripping Solution (Millipore, Billerica, MA, USA). The expression level of genes was then evaluated by using ImageJ software (ver. 1.43; http://rsbweb.nih.gov/ij/index.html).

### Transfection

Pre-miR miRNA precursor and negative control (Applied Biosystems) were used in the gain-of-function experiments. RAC1, EGFR and EP300 siRNA (Sigma) and negative control siRNA (D-001810-10; Thermo Fisher Scientific, Waltham, MA, USA) were used in the loss-of-function experiments. PC3 and DU145 cells were transiently transfected using Lipofectamine 2000 transfection reagent (Invitrogen), according to the manufacturer’s recommendations.

### Cell Proliferation, Migration, and Invasion Assays

Cell proliferation was measured using a CellTiter 96 AQueous One Solution Cell Proliferation Assay (MTS) (Promega, Madison, WI, USA) performed according to the manufacturer’s instructions. Cell proliferation was determined by absorbance measurements at 490 nm using SpectraMAX 190 (Molecular Devices Co., Sunnyvale, CA, USA). Cell migration activity was evaluated by a wound-healing assay. Cells were plated in six-well dishes, and the cell monolayers were scraped using a P-20 micropipette tip. The width of the initial gap (0 h) and the residual gap 6, 12 and 24 hours after wounding were calculated from photomicrographs. A cell invasion assay was carried out using modified Boyden Chambers consisting of transwell-precoated Matrigel membrane filter inserts with eight micron pores in 24-well tissue culture plates (BD Biosciences, Bedford, MA, USA). Minimum essential medium containing 10% FBS in the lower chamber served as the chemoattractant, as described previously [55]. All experiments were performed in triplicate.

### In vivo Tumor Growth

All animal care was in accordance with the guidelines of the San Francisco Veterans Affairs Medical Center and the study was approved by the San Francisco VA IACUC (Protocol number: 11-008-01). Animal users have completed training programs to handle and work with mice through AALAS (American Association for Laboratory Animal Science) prior to animal experiments. For the subcutaneous xenograft mouse model, DU145 cells (2.5×10^6^) that were transiently transfected with miR-574-3p or miR-control were suspended in 50 µL RPMI 1640 medium and were subcutaneously injected into female nude mice (strain BALB/c nu/nu; Charles River Laboratories, Inc., Wilmington, MA, USA, 5 weeks old). A total of 8 nude mice (4-miR-574-3p, 4-miR-control) were used and tumor growth was examined over the course of 35 days. Tumor volume was calculated on the basis of width (x) and length (y): x^2^y/2, where x<y.

### Apoptosis Assays

Fluorescence-activated cell-sorting (FACS) analysis for apoptosis was done 96 hours post-transfection, using Annexin V-FITC/7-AAD Kit (Beckman Coulter, Brea, CA, USA), according to the manufacturer's protocol. Stained cells were immediately analyzed with a flow cytometer (Cell Lab Quanta SC; Beckman Coulter).

### Identification of miR-574-3p Regulated Target Genes and Bioinformatic Analysis

To search for genes regulated by miR-574-3p, we used TargetScan algorism (release 6.2, http://www.targetscan.org/). To identify the biological processes or pathways potentially regulated by miR-574-3p, we performed GeneCodis analysis (http://genecodis.dacya.ucm.es/) using all of the candidate genes. Then, to identify networks among the miRNAs and their target genes, we analyzed and characterized those genes in KEGG (Kyoto Encyclopedia of Genes and Genomes) pathway categories. These data were used to examine miRNA-regulated molecular networks in human cells. We performed gene expression analyses of all candidate genes involved in each of the pathways using microarray expression data approved by the GEO and were assigned GEO accession numbers (GSE29079). In the Affymetrix Human Exon 1.0 ST Array (Affymetrix, Santa Clara, CA, USA) datasets, we examined 47 PCa tissues and 47 normal prostate tissues, all of which were collected from patients who had not been exposed to neo-adjuvant radio-, cytotoxic- or endocrine therapy before the operation. The data was normalized and analyzed with the GeneSpring (Agilent Technologies, Santa Clara, CA, USA). Statistical analyses were conducted using the Mann Whitney U-test with cut-off P<0.05.

### Plasmid Construction and Dual-luciferase Reporter Assays

For 3′ UTR luciferase reporter assay, PmirGLO Dual-Luciferase miRNA Target Expression Vector was used (Promega). The oligonucleotide sequences (wild-type) used are shown in Table S1. We also constructed mutated oligonucleotides for each of the wild-type oligonucleotides (Table S1). In a total volume of 25 µl, 1 µl each of 100 µM forward and reverse oligonucleotide, 2.5 µl of 10× annealing buffer (100 mM Tris–HCl, pH 7.5, 1 M NaCl and 10 mM ethylenediaminetetraacetic acid) and 20.5 µl water were incubated at 95°C for 3 min and then placed at 37°C for 15 min. The oligonucleotides were ligated into the PmeI–XbaI site of pmirGLO Dual-Luciferase miRNA Target Expression Vector. For 3′ UTR luciferase assay, PCa cells were co-transfected with Pre-miR miRNA precursor and pmirGLO Dual-Luciferase miRNA Target Expression Vectors using Lipofectamine 2000 (Invitrogen) and X-tremeGENE HP DNA Transfection Reagent (Roche Diagnosis, Basel, Switzerland, USA) according to the manufacturer’s instructions. Luciferase reporter assay was performed using the Dual-Luciferase Reporter Assay System (Promega) 24 hours after transfection. Firefly luciferase activities were normalized with Renilla luciferase. We also included basic vector containing no insert as a mock control.

### Statistical Analysis

The relationship between two variables and the numerical values obtained by real-time RT-PCR were analyzed using the nonparametric Mann-Whitney U test. All analyses were performed using Expert StatView (version 4, SAS Institute Inc., Cary, NC, USA). Data are shown as mean values ± standard error. P values of <0.05 were regarded as statistically significant.

## Supporting Information

Table S1 **Primer oligonucleotide sequences (wild-type and mutated).**
(DOC)
